# Case Report of a Hidden Intruder—Extremely Rare Presentation of Hydatidosis as a Nuchal Tumoural Mass

**DOI:** 10.3389/bjbs.2024.12446

**Published:** 2024-04-08

**Authors:** Dragoș F. Voicu, Constantin Popazu, Delia Mihaela Râșnoveanu, Daniela Mihalache, Alexandra Toma

**Affiliations:** ^1^ Faculty of Medicine and Pharmacy, Dunarea de Jos University of Galati, Galati, Romania; ^2^ Emergency Clinical County Hospital of Braila, Braila, Romania

**Keywords:** cervical hydatid cyst, primary hydatidosis, hydatid disease, echinococcus granulosus infection, hydatid cyst

## Abstract

**Introduction:** The parasitic tapeworm impersonated by the larvae of *Echinococcus granulosus* represents the aetiology of the hydatid pathology. The predilect site of invasion is the liver, but there are other cases of different localization all over the body, regardless of the type of invaded tissue. Soft tissue hydatidosis can be a real challenge for the clinician in terms of the diagnosis, and it might generate various complications such as anaphylactic shock. The aim of the present work is to illustrate a unique case of primary hydatidosis located in the nuchal region.

**Case Report:** We report the case of a 68-year-old male patient, a zootechnic, who presented at the hospital with a tumoural mass (dimension: about 12/10 cm) located in the nuchal region. The complex approach needed consisted of surgical therapy along with histopathological confirmation of the diagnostic and antiparasitic medication, which led to a complete recovery with a low probability of recurrence.

**Discussion:** Encounters with patients with primary soft tissue hydatidosis are exceptionally rare, but the surgeon must take into consideration this clinical diagnosis, especially for patients located in an endemic region with occupations that might have exposed the patient to this type of parasite.

## Introduction

In 1821, Bremser documented the inaugural case of a hydatid cyst, heralding the advent of scientific inquiry into this parasitic affliction [1]. Hydatid cysts result from infestation by the cestode parasite *Echinococcus granulosus*, leading to the condition known as hydatidosis or echinococcosis [2]. This pathology is a prominent global zoonotic infection that significantly impacts public health and agriculture. Echinococcosis, which can manifest between the ages of 6 and 55, includes both symptomatic and asymptomatic cases and is identified through screening [3].

Transmission of echinococcosis occurs via the digestive route, following ingestion of parasitic eggs present in the faeces of infected animals. The life cycle of the parasite involves canids—such as dogs, foxes, and wolves—as definitive hosts and herbivores—such as sheep, cattle, and goats—as intermediate hosts. Humans may become accidental intermediate hosts through consumption of contaminated food or water or via direct contact with infected animals. Once ingested, the parasite’s embryo enters the intermediate host’s entero-hepatic bloodstream, emphasising the disease’s prevalence in regions with intensive sheep farming.

In human hosts, 50%–75% of hydatid cysts develop in the liver, 25% in the lungs, and 5%–10% are disseminated through the arterial system, potentially affecting any organ, including the kidney, spleen, pancreas, brain, muscles, and bones [2].

The clinical manifestations of hydatidosis vary with the cyst’s location and size, often remaining asymptomatic until significant growth induces symptoms or complications. Ultrasound (US) and computed tomography (CT) scans are the principal diagnostic tools.

The occurrence of hydatid cysts in the nuchal region is exceedingly rare [4, 5], with most cases affecting the liver and lungs. Few reports exist of hydatidosis in unconventional locations, such as the orbit, spinal cord, or soft tissues, possibly resulting from haematogenous migration or direct extension from an adjacent organ [6].

This case report aims to heighten awareness of the potential for hydatidosis to present as a tumoural mass in the nuchal region, highlighting the need to include this parasitic infection in differential diagnoses, especially in areas where the disease is endemic. Prompt recognition and effective management are crucial to averting complications, minimizing morbidity, and enhancing patient outcomes.

## Case Description

### Clinical Presentation

**FIGURE 1:**
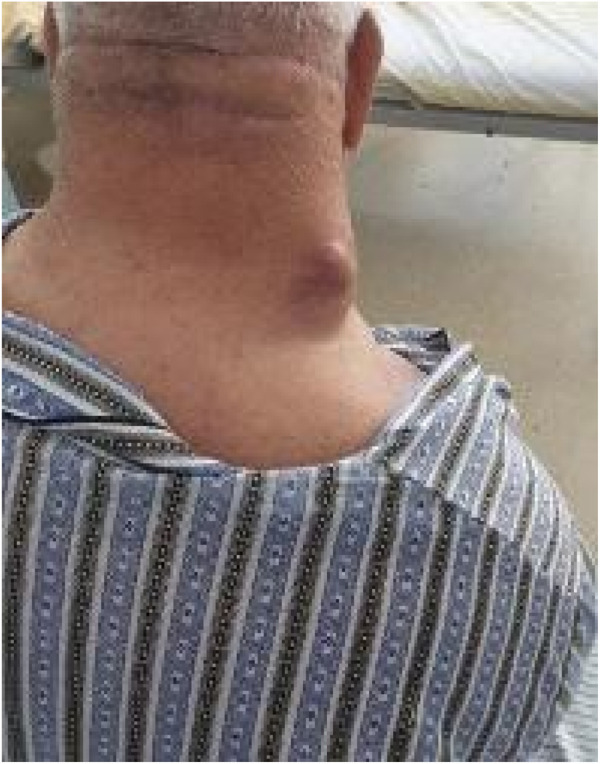
Clinical aspect of the nuchal region mass.

A 68-year-old male, professionally engaged in zootechnics as a sheep breeder, visited the hospital due to a progressively growing mass in the right lateral nuchal region. The patient reported experiencing sporadic pain and discomfort within the affected area over the preceding 6 months, which resulted in an aesthetically displeasing appearance of the region (Figure 1).

Furthermore, the patient did not report any associated symptoms such as fever, weight loss, or respiratory distress. He specifically denied any history of trauma or prior surgical interventions in the nuchal region. However, he did recall an incident from 6 years prior when the mass was smaller and had been aspirated, yielding clear fluid. Following this aspiration, the mass gradually increased in size over time.

Upon physical examination, a palpable mass was identified in the nuchal region. The mass exhibited firm consistency and well-defined margins. Notably, the overlying skin displayed signs of inflammation, though no discharge was observed. Examination of the surrounding area revealed the absence of palpable lymph nodes.

The patient’s medical history revealed no remarkable findings, and there was no known exposure to echinococcosis or other parasitic infections.

### Diagnostic Workup

To characterise the nature of the nuchal mass, a series of imaging studies were conducted. A computed tomography (CT) scan of the nuchal region revealed a distinct encapsulated lesion measuring approximately 12/10 cm. The lesion exhibited heterogeneous internal content with areas of low and high attenuation along with peripheral calcifications. Importantly, adjacent structures such as the cervical vertebrae, muscles, and blood vessels appeared unremarkable, showing no signs of infiltration or compression.

Based on the radiological findings, a provisional diagnosis of a tumoural mass, potentially of infectious aetiology, was made. Consequently, surgical excision of the nuchal mass was planned to obtain a definitive diagnosis and provide appropriate therapeutic intervention.

### Surgical Intervention and Histopathological Examination

Under general anaesthesia, the patient underwent surgical excision of the nuchal mass. Intraoperatively, the mass was found to be well-encapsulated and adherent to the surrounding tissues. Meticulous dissection was carried out to avoid rupture and dissemination of any infectious material.

### The Excised Specimen was Submitted for Histopathological Examination

**FIGURE 2:**
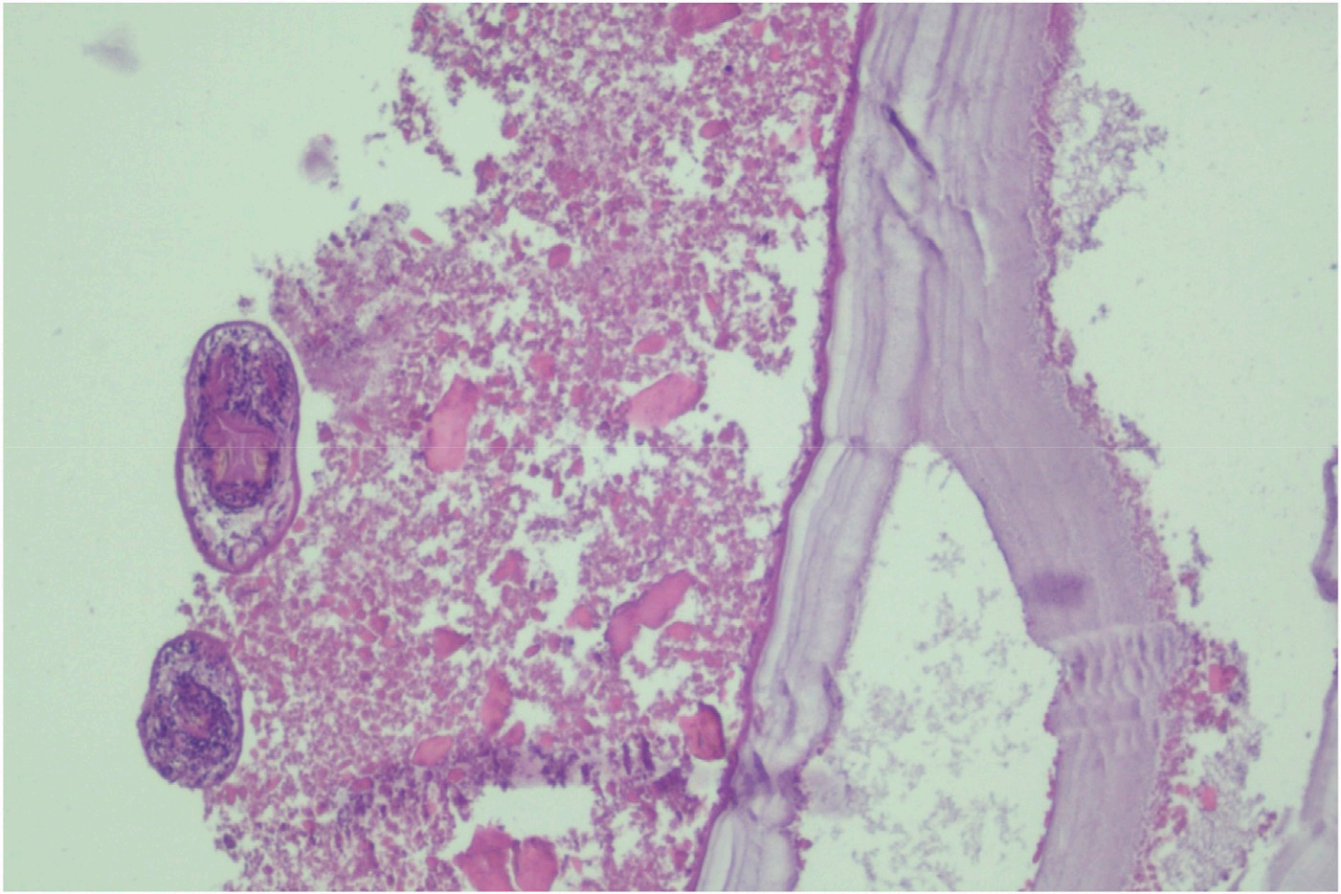
Histopathological specimen illustrating inner germinal and lamellated layers of cyst wall.

**FIGURE 3:**
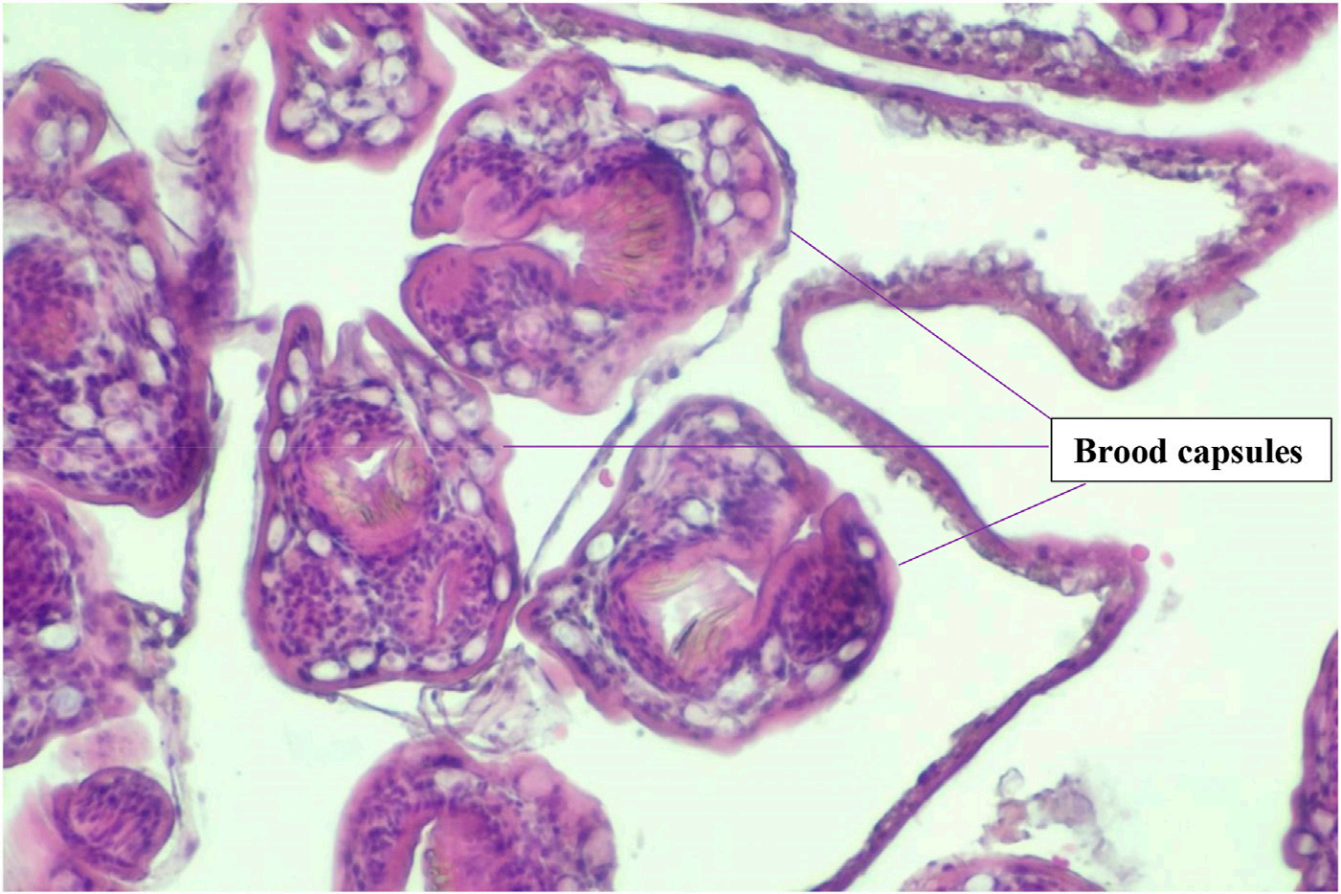
Histopathologic findings of Brood Capsules in the surgical specimen.

Microscopic analysis, as depicted in Figures 2, 3, revealed the presence of characteristic hydatid cyst structures. These included an outer laminated layer and an inner germinal layer along with numerous protoscolices. Additionally, a marked foreign body giant cell reaction was observed. These histological findings provided conclusive evidence confirming the diagnosis of hydatidosis.

### Postoperative Course and Follow-Up Regimen

Following the surgical intervention, the patient underwent a comprehensive postoperative care regimen. He was closely monitored for any signs of complications or recurrence of symptoms. Additionally, albendazole therapy was initiated promptly to prevent potential recurrence and control any residual parasitic activity.

Regular follow-up appointments were scheduled to assess the patient’s progress and monitor for any signs of recurrence or complications. Imaging studies, including CT scans and ultrasound examinations, were conducted periodically to evaluate the status of the surgical site and ensure the absence of residual cystic lesions.

Throughout the follow-up period, the patient remained asymptomatic, with no evidence of recurrence or complications noted during clinical examinations. The albendazole therapy was well-tolerated, with no reported adverse effects.

At each follow-up visit, the patient received comprehensive counselling regarding the importance of adherence to medication as well as preventive measures to minimise the risk of re-infection. He was advised to maintain good hygiene practices and avoid exposure to potentially contaminated environments.

The patient’s compliance with the prescribed treatment regimen and follow-up appointments was exemplary, facilitating optimal management and ensuring favourable outcomes. With continued monitoring and adherence to medical therapy, the patient’s long-term prognosis remained favourable, with the expectation of sustained remission and preservation of his overall health and wellbeing.

## Discussion

Hydatidosis, attributed to parasitic infestation by *Echinococcus granulosus* represents a formidable global public health challenge that is intricately intertwined with human and livestock welfare. While the liver and lungs serve as the primary sites of predilection, the emergence of hydatid cysts in ectopic locations remains an enigmatic rarity. The striking presentation of a hydatid cyst within the nuchal region, as delineated in this case report, evokes profound curiosity regarding the underlying mechanisms governing parasite dissemination and the intricate dynamics of host–parasite interactions.

In the backdrop of its oft-indolent course, characterised by a paucity of overt symptoms, the conspicuous appearance of a discernible tumoural mass in the nuchal region precipitated an urgent impetus for comprehensive diagnostic exploration. Differential diagnostic considerations, spanning a broad spectrum encompassing both neoplastic and non-neoplastic aetiologies, necessitated an exhaustive diagnostic odyssey to unravel the precise aetiology of the lesion.

The pivotal role of advanced imaging modalities, including computed tomography (CT), magnetic resonance imaging (MRI), and ultrasound, assumes paramount significance in unravelling the intricate morphological attributes of hydatid cysts. These modalities not only afford a nuanced understanding of cystic morphology but also inform surgical strategy, thus underpinning the cornerstone of precise diagnosis and therapeutic intervention. Moreover, adjunctive serological assays, such as enzyme-linked immunosorbent assay (ELISA), serve as indispensable adjuncts in augmenting diagnostic precision, particularly in instances where clinical and radiological findings yield equivocal results.

Surgical excision emerges as the quintessential therapeutic paradigm for hydatid cysts, with the primary objective being complete cyst eradication to obviate the perils of cystic rupture and ensuing complications. The successful execution of surgical procedures necessitates meticulous intraoperative manoeuvres to mitigate the risk of inadvertent dissemination. In instances where complete excision proves elusive, judicious consideration of partial resection augmented by adjunctive medical therapy assumes paramount significance in optimising therapeutic outcomes.

The postoperative management landscape of hydatidosis encompasses the judicious administration of anthelmintic agents, notably albendazole or mebendazole, to forestall disease recurrence and preclude the dissemination of parasitic elements. The demonstrated efficacy of postoperative medical therapy underscores its pivotal role in fortifying the therapeutic armamentarium against hydatidosis.

Furthermore, the imperative of vigilant postoperative surveillance, fortified by periodic imaging evaluations, cannot be overstated in the enduring management paradigm of hydatidosis. Such surveillance endeavours serve as a sentinel against disease resurgence or the emergence of *de novo* lesions, thereby facilitating timely intervention and optimising patient outcomes.

In summation, this case report serves as a poignant testament to the myriad clinical manifestations of hydatidosis and underscores the imperative of vigilance in diagnostic deliberations surrounding tumoural masses, even in atypical anatomical locales. The continuum of research endeavours aimed at unravelling the enigmatic intricacies of this zoonotic malady is imperative for refining therapeutic modalities and optimising clinical outcomes in the pursuit of global hydatidosis control.

## Conclusion

In conclusion, this case report underscores the mysterious characteristics of hydatidosis, its ability to manifest in atypical anatomical locations, and the pivotal role of diagnostic, surgical, and therapeutic approaches in its management. It exemplifies the advancements in medical imaging, diagnostic methodologies, and surgical techniques that are integral to the effective treatment of such uncommon and demanding cases.

Furthermore, it emphasises the significance of continuous research and education in the realm of parasitology to enhance comprehension and mitigation of the intricate facets associated with this zoonotic ailment.

## Data Availability

The raw data supporting the conclusion of this article will be made available by the authors, without undue reservation.
